# Activity interventions to improve the experience of care in hospital for people living with dementia: a systematic review

**DOI:** 10.1186/s12877-020-01534-7

**Published:** 2020-04-10

**Authors:** Ilianna Lourida, Ruth Gwernan-Jones, Rebecca Abbott, Morwenna Rogers, Colin Green, Susan Ball, Anthony Hemsley, Debbie Cheeseman, Linda Clare, Darren Moore, Chrissy Hussey, George Coxon, David J. Llewellyn, Tina Naldrett, Jo Thompson Coon

**Affiliations:** 1 NIHR Applied Research Collaboration (ARC), Evidence Synthesis Team, PenARC, University of Exeter Medical School, St Luke’s Campus, University of Exeter, Exeter, EX1 2LU UK; 2 Health Economics Group, University of Exeter Medical School, St Luke’s Campus, University of Exeter, Exeter, EX1 2LU UK; 3 Health Statistics Group, PenARC, University of Exeter Medical School, College of Medicine and Health, St Luke’s Campus, University of Exeter, Exeter, EX1 2LU UK; 4 Royal Devon and Exeter NHS Foundation Trust, Barrack Road, Exeter, EX2 5DW UK; 5 Centre for Research in Aging and Cognitive Health, University of Exeter Medical School, St Luke’s Campus, University of Exeter, Exeter, EX1 2LU UK; 6 Graduate School of Education, College of Social Sciences and International Studies, St Luke’s Campus, University of Exeter, Exeter, EX1 2LU UK; 7 Hospiscare, Dryden Road, Exeter, EX2 5JJ UK; 8 Devon Care Kitemark, Pottles Court, Days-Pottles Lane, Exminster, Exeter, EX6 8DG UK; 9 Mental Health Research Group, University of Exeter Medical School, St Luke’s Campus, University of Exeter, Exeter, EX1 2LU UK; 10 The Alan Turing Institute, London, UK

**Keywords:** Dementia, Hospital, Acute care, Experience, Non-pharmacological interventions, Systematic review

## Abstract

**Background:**

An increasingly high number of patients admitted to hospital have dementia. Hospital environments can be particularly confusing and challenging for people living with dementia (Plwd) impacting their wellbeing and the ability to optimize their care. Improving the experience of care in hospital has been recognized as a priority, and non-pharmacological interventions including activity interventions have been associated with improved wellbeing and behavioral outcomes for Plwd in other settings. This systematic review aimed at evaluating the effectiveness of activity interventions to improve experience of care for Plwd in hospital.

**Methods:**

Systematic searches were conducted in 16 electronic databases up to October 2019. Reference lists of included studies and forward citation searching were also conducted. Quantitative studies reporting comparative data for activity interventions delivered to Plwd aiming to improve their experience of care in hospital were included. Screening for inclusion, data extraction and quality appraisal were performed independently by two reviewers with discrepancies resolved by discussion with a third where necessary. Standardized mean differences (SMDs) were calculated where possible to support narrative statements and aid interpretation.

**Results:**

Six studies met the inclusion criteria (one randomized and five non-randomized uncontrolled studies) including 216 Plwd. Activity interventions evaluated music, art, social, psychotherapeutic, and combinations of tailored activities in relation to wellbeing outcomes. Although studies were generally underpowered, findings indicated beneficial effects of activity interventions with improved mood and engagement of Plwd while in hospital, and reduced levels of responsive behaviors. Calculated SMDs ranged from very small to large but were mostly statistically non-significant.

**Conclusions:**

The small number of identified studies indicate that activity-based interventions implemented in hospitals may be effective in improving aspects of the care experience for Plwd. Larger well-conducted studies are needed to fully evaluate the potential of this type of non-pharmacological intervention to improve experience of care in hospital settings, and whether any benefits extend to staff wellbeing and the wider ward environment.

## Background

Demographic ageing is associated with increased rates of acute general hospital admissions for older people with multiple comorbidities and complex care needs [1]. The estimated 850,000 people living with dementia (Plwd) in the UK and over 46 million people worldwide are over-represented in this inpatient population: approximately 25% of hospital beds are occupied by Plwd [2, 3] who often have several additional long-term conditions (e.g. heart disease, diabetes) [4]. Those admitted to hospital with dementia experience complications and adverse outcomes including longer length of stay, greater mortality and increased risk of institutionalization post-discharge compared to those without dementia [5, 6]. While 20% of hospital admissions of Plwd are potentially preventable [7] some unplanned admissions are unavoidable, and it is important that hospital care supports the needs of those affected by dementia. Hospital settings with high noise levels and unfamiliar surroundings can be overwhelming and confusing for Plwd, impacting their wellbeing and the ability to optimize their care. In addition, what happens in hospitals can have a profound and permanent effect on individuals and their families, not only in terms of their inpatient experience, but also their ongoing health and the decisions that are made about their future [8, 9].

Patient experience is one of the three pillars of quality of care along with clinical effectiveness and safety [10]. Improving the experience of care for Plwd in the hospital setting is a key component of dementia research priorities and national policies in many countries including the UK [11, 12]. In 2011, the Royal College of Nursing (RCN) published five principles for improving dementia care in hospital settings: staff, partnership, assessment, individualized care and environments (SPACE) [13, 14]. The RCN SPACE principles have helped promote a key objective of the UK national dementia strategy, to improve hospital care for Plwd [15]. Whilst these principles have been detailed and set out as a resource for those involved in care, providing effective acute care services to Plwd remains an ongoing challenge [16, 17]. National health care organizations have developed guidelines for dementia care interventions, including recommendations for practitioners to offer a range of activities to promote wellbeing which are tailored to the person’s preferences [18, 19]. Numerous media reports have described interventions to improve the experience of care for Plwd in hospitals including services and activities such as dementia gardens, painting, listening to music or the introduction of tools to provide relief from restlessness [20–22]. However, most of this evidence is anecdotal and is not accompanied by evaluation of the interventions.

Reviews of studies on the effects of a broad range of non-pharmacological interventions on Plwd have reported that activity programs, such as music and other sensory interventions, are associated with improved activities of daily living, cognition, quality of life, anxiety and depression in Plwd [23–25]. There are also a number of systematic reviews indicating some benefit of activity interventions on behavioral and psychological symptoms of dementia (BPSD) [26–29]. However, some of these reviews do not focus on activity interventions or have not identified any research conducted in the hospital setting [23, 26, 30], and others have not used robust systematic methods [29, 31]. Furthermore, how activity interventions affect the experience of care has not been evaluated. Examining experience of care may benefit current hospital care practice, resulting in better care for those with dementia and support for those involved in their care, as well as highlighting areas in which we have limited understanding of how to achieve best practice.

Therefore, our aim was to synthesize the available evidence evaluating the effectiveness of activity-based interventions (encompassing those described as ‘activities’ or ‘activity programs’) to improve the experience of care for Plwd while in hospital. This systematic review is part of a larger project of three linked systematic reviews exploring the perspectives of experience of care in hospital, and evaluating the effectiveness and cost-effectiveness of interventions to improve experience of care in hospital for Plwd, their family and/or friends, and hospital staff caring for them (Health Services and Delivery Research programme of the National Institute for Health Research project: 16/52/52, PROSPERO registration CRD42018086013). The project integrated end-user involvement throughout the reviews in the form of input and feedback from a project advisory group consisting of public, clinical and academic topic experts.

## Methods

### Identification of studies

#### Search strategy

Search terms were selected to cover dementia, hospital settings and names of interventions which were informed by the qualitative reviews of the larger linked project and through consultations with the project advisory group. The following databases were searched on the 9th and 10th May 2018 and updated on 2nd October 2019: MEDLINE, EMBASE, PsycINFO, HMIC, Social Policy & Practice (via OvidSp), Cochrane Database of Systematic Reviews, CENTRAL, NHS EED, DARE and the HTA database (via the CRD Database), CINAHL (via EBSCOhost), BNI (via ProQuest), and SSCI and the Conference Proceedings Citation Index (via Web of Science) and Proquest Dissertations & Theses Global. The search strategy as designed in MEDLINE and adapted for the other databases is available in Additional file 1. Forwards and backwards citation chasing was carried out for all included studies.

#### Inclusion and exclusion criteria

The following inclusion and exclusion criteria were used to determine eligibility:

**Population:** people with cognitive impairment or dementia. Studies including older people with delirium/confusion or other physical or mental health conditions were included if data for Plwd were retrievable and represented ≥50% of the sample.

**Intervention:** any intervention delivered to Plwd aiming to improve their experience of care in hospital focusing on active participation of Plwd in occupational, social and cultural activities.

**Comparator:** any control or comparator.

**Setting:** any hospital setting, including the process of transition into and out of hospital.

**Outcomes:** our project advisory group (dementia researchers, nursing, medical, physiotherapy staff, care home activity coordinator, palliative care practitioner, current and previous carers of Plwd) defined experience of care as ‘the extent to which a person perceives that needs arising from physical and emotional aspects of being ill are met’. Therefore, primary outcomes included any outcome describing the experience of or outcome of care. Behavioral indicators (e.g. agitation) were included as secondary outcomes where studies fulfilled all inclusion criteria.

**Study design:** all quantitative study designs reporting comparative data (i.e. with control group or pre-post comparison), prioritizing evidence from more robust study designs in the synthesis where possible.

#### Study selection

Records retrieved from the database searching were imported to Endnote software v.X8 (Clarivate Analytics, USA) for screening. Titles and abstracts for each record were assessed independently by two reviewers (IL, RA, MR or SD) against the inclusion criteria. Disagreements between reviewers were resolved through discussion with the involvement of a third reviewer where necessary (RGJ, RA, MR or JTC). The full texts of potentially relevant records were obtained through web searching, the University of Exeter online library or the British Library. Full texts were assessed in the same way by two reviewers (IL, SD) and disagreements were resolved through discussion with a third (RGJ, RA, MR or JTC).

#### Data extraction

Data were extracted in Microsoft Excel (2013) by one reviewer (IL) and checked by a second (RGJ). Extracted data included details on study author, year and publication type, country, study design, sample size and participant characteristics at baseline, hospital setting details, dementia status/assessment of participants, reason for hospital admission, intervention name, recipient and provider, comparator, follow-up duration, outcomes and method of assessment, type of statistical analysis and results (means, standard deviations, *p*-values). Additional intervention details were extracted to enhance understanding of intervention content and aims, and facilitate description of intervention characteristics using items from the Template for Intervention Description and Replication (TIDieR) checklist [32]. Individual intervention components based on descriptions provided in the studies were also extracted to inform the synthesis.

#### Quality assessment

All studies were critically appraised using the Effective Public Health Practice Project Quality Assessment tool [33]. Study quality is rated based on six components, namely selection bias, study design, confounders, blinding, data collection methods, and withdrawals and drop-outs. Individual component ratings count towards a global rating of ‘strong’, ‘moderate’ or ‘weak’ quality for each study. Quality assessment was conducted independently by two reviewers (IL, RGJ) with recourse to a third in case of disagreement (RA). The tool was used to assess study quality and not to exclude studies.

#### Categorization of interventions and outcomes

Following data extraction by one reviewer (IL), intervention components were broadly categorized based on similarities in concept and content. Interventions could consist of one or more components which described key features of the intervention content, such as specialist capacity added to assist with the activity, e.g. a musician. Initial components were refined after discussion with a second reviewer (RGJ) and were subsequently discussed with the core review team.

Outcomes used to assess the effectiveness of interventions were categorized after mapping the underlying constructs measured by the tools used in included studies. The categories were developed by one reviewer (IL) locating the original items where possible through online resources to clarify the measured constructs. Outcome clusters and any links among outcomes were then discussed with a second reviewer (RGJ), refined, and shared with the core review team. Outcome categories were used to organize findings for similar outcomes, and to help determine whether results for outcomes measuring comparable constructs were suitable for meta-analysis. The outcome categories, and measures used in each of the included studies are shown in Additional file 2.

#### Data analysis and synthesis

Findings were tabulated using sample sizes, means and standard deviations (SDs) or range. Effectiveness was assessed based on differences in means between intervention and control groups at post-test for the one randomized controlled trial and between pre- and post-intervention measurements for non-randomized uncontrolled studies. Effect sizes were calculated to assess differences and aid interpretation of findings using standardized mean differences (SMDs), which represent the difference between the means in the two groups divided by their pooled standard deviation (Cohen’s *d*). The SMDs and 95% confidence intervals (CIs) were calculated using the Campbell Collaboration online calculator accessed at: http://www.campbellcollaboration.org/escalc/html/EffectSizeCalculator-SMD1.php. Cohen’s guidelines [34] were used to interpret the effect sizes as follows: small ≥0.20 and < 0.50, medium ≥0.50 and < 0.80, and large ≥0.80. SMDs for the uncontrolled before-after studies were calculated on the assumption of paired pre-post intervention comparisons. Mean differences (non-standardized) with 95% CIs and *p*-values were also calculated for each outcome across studies.

Meta-analysis was considered feasible for studies that shared the same study design, outcome category, and included a similar participant group. Additionally, we required paired pre-post comparisons for the meta-analysis of outcomes of uncontrolled before-after studies. However, the data available were not sufficient for meta-analysis and therefore the effectiveness of interventions to improve experience of care was described through a narrative synthesis approach. After summarizing study and intervention characteristics, findings are presented in narrative form according to the identified outcome categories. The narrative synthesis is accompanied by tables with raw data and effect sizes where calculable.

## Results

### Study selection

**Fig. 1:**
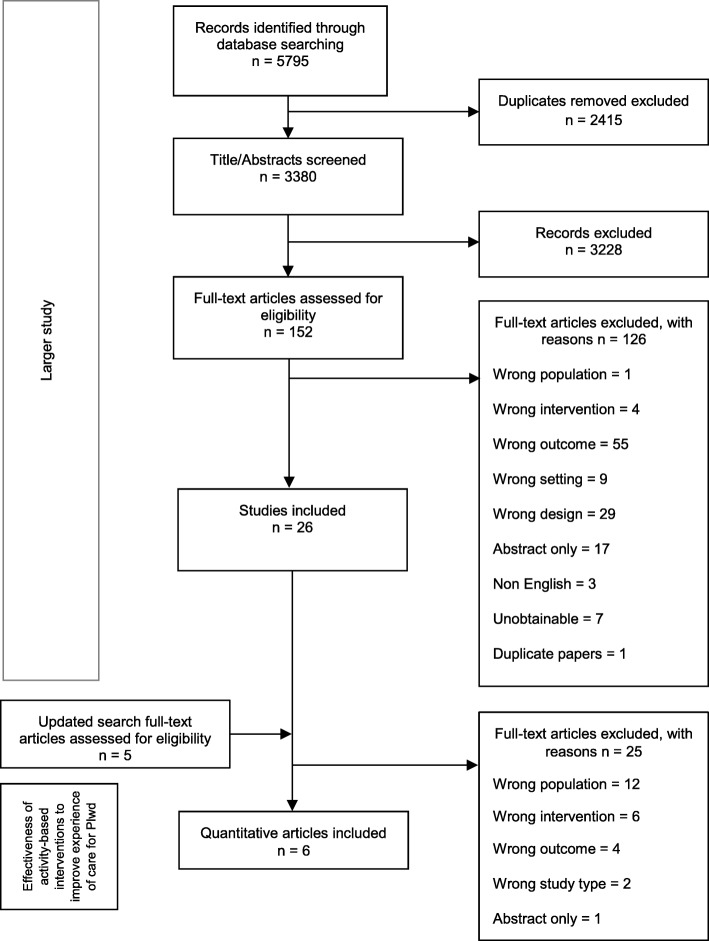
PRISMA flow diagram showing study screening and selection process

The Preferred Reporting Items for Systematic Reviews and Meta-Analyses (PRISMA) flowchart [35] shown in Fig. 1 summarizes the study selection process. After deduplication, a total of 3380 records were screened at title and abstract stage resulting in 152 records for full text review to further assess eligibility. Of these, seven were unobtainable and 126 were excluded for reasons shown in Fig. 1. The most common reasons for exclusion were not including outcomes measuring experience of or outcome of care (44%, *n* = 55), and not being a quantitative study design reporting comparative data (23%, *n* = 29). For 17 records only abstracts were available and therefore there was insufficient information to determine inclusion. Updated searches resulted in five new relevant studies that were screened at full text and then excluded (two did not report experience of care outcomes, two were not quantitative studies with comparative data, and one was a conference abstract and currently unpublished). No new studies were found through citation chasing.

### Study and sample characteristics

Six studies met the inclusion criteria and were included in the synthesis. Table 1 reports on the study and participant characteristics. Of the six included studies one was a randomized controlled trial (RCT) [36], two were time series studies [37, 38], two were uncontrolled before-after studies [39, 40], and one was a prospective cohort study [41]. All included studies were journal articles and published between 2009 and 2018. Studies were conducted in USA (*n* = 2 [36, 37];), UK (*n* = 2 [40, 41];), Singapore (*n* = 1 [39];), and Switzerland (*n* = 1 [38];).

**Table 1:** Summary of characteristics of activity-based intervention studies to improve experience of care in hospital for Plwd

Study, country	Design	Population characteristics at baseline	Dementia status/ assessment	Reason for hospital admission; type of ward/facility	Intervention	Comparator	Outcomes	Study duration
DiNapoli et al. (2016) [36] USA	RCT	*n* = 52 Plwd (I: 26, C: 26), mean age: 70.6, 60% women	Mild to moderate cognitive impairment as indicated by a score of 15–27 on the Saint Louis University Mental Status Examination	Involuntarily committed to receive care as a result of displaying inappropriate behaviours or disturbing the peace; Psychogeriatric (geriatric psychiatry facility)	Individualized social activities intervention to improve QoL of cognitively impaired geriatric patients	Usual care including scheduled psychoeducational groups, pharmaco-therapy, and social work consults provided by the facility	QoL, behaviour	up to 2 weeks
Gitlin et al. (2016) [37] USA	TS	*n* = 20 Plwd, mean age: 77.6, 60% women	Patients with a dementia diagnosis	Behavioural disturbances; Psychogeriatric (medical behavioural unit)	Tailored Activity Program for Hospitals (TAP-H) to improve engagement in Plwd admitted for behavioural disturbances	Standardised activity session at baseline	Engagement, mood	unclear
Weber et al. (2009) [38] Switzerland	TS	*n* = 76 Plwd, mean age: 77.5, 49% women	Clinical diagnosis of dementia according to ICD-10 criteria. Dementia severity assessed using the Clinical Dementia Rating Scale	Behavioural disturbances; Psychiatric day hospital	Psychotherapeutically-oriented day hospital program for Plwd with BPSD	Admission as baseline time point	Engagement (therapeutic progress), behaviour	12+ months
Cheong et al. (2016) [39] Singapore	BA	n = 25 Plwd/delirium, mean age: 86.5, 60% women	Dementia diagnosed by geriatricians according to DSM-IV; delirium ascertained with the Confusion Assessment Method (CAM)	Not specified; Acute care	Creative music therapy sessions for Plwd and/or delirium to improve wellbeing	(pre-intervention)	Engagement, mood	3 months
Daykin et al. (2017) [40] UK	BA	n = 20 Plwd, aged > 80, 65% women	Patients with a dementia diagnosis	Not specified; Acute care	Inclusive participatory music activity to support wellbeing of Plwd	(pre-intervention)	Behaviour	10 weeks
Windle et al. (2018) [41] UK	PC	*n* = 23 Plwd, mean age 81.4, 52% women	Patients with a dementia diagnosis or evidence of age-related memory impairment. Dementia severity assessed using the Clinical Dementia Rating Scale	Not specified; NHS assessment units	Visual arts programme to improve wellbeing and social behaviours of Plwd	Unstructured social activity with no arts activities at baseline	Wellbeing, QoL, engagement (communication)	6 months

*BA* Before-after study (pre-post), *C* Controls, *DSM* Diagnostic and Statistical Manual of Mental Disorders, *ICD-10* International Classification of Diseases, 10th revision, *I* Intervention, *NHS* National Health Service, *PC* Prospective cohort, *Plwd* People living with dementia, *QoL* Quality of life, *RCT* Randomised controlled trial, *TS* Time-series

Studies included a total of 216 Plwd. The mean age of Plwd ranged from 70.6 to 86.5 years and the percentage of female participants ranged from 49 to 65%. Two studies described Plwd as previously diagnosed with dementia without detailed information related to dementia status or assessment within the study [37, 40]. Two studies [38, 39] reported a dementia diagnosis according to established diagnostic criteria, one study reported dementia severity assessed using the Clinical Dementia Rating scale [41], and one study reported a cognitive test score cut-off for mild cognitive impairment as part of the inclusion criteria [36]. In one of the studies selection criteria included patients with dementia with or without delirium [39] although further details about the diagnoses of recruited Plwd were not reported.

Three of the studies did not specify the reason for hospital admission, and the remaining studies reported behavioral disturbances (e.g. aggression, agitation) [37, 38] or involuntary admission to a psychiatric facility as a result of disturbing the peace or displaying inappropriate behaviors [36]. In terms of study setting, two were conducted in acute care wards, one in National Health Service (NHS) assessment units, one in a psychiatric day hospital [38], and two in psychogeriatric units [36, 37]. Study duration varied across studies but was generally short and ranged from 2 weeks to 12 months. Studies included a number of outcomes collectively described as aspects of wellbeing, with categories for engagement, mood, quality of life and wellbeing.

### Quality assessment

One study, the RCT, [36] received a ‘strong’ global quality rating, with the remaining studies rated as ‘weak’ [37–41] (see Table 2). The RCT reported 1:1 allocation using block randomization, and reliable and valid assessment tools. Selection bias was rated as moderate following a sample of involuntarily committed participants (by probate court). Assessors were blind to participant group allocation but it was unclear whether participants themselves were aware of the research question. Withdrawals and drop-outs were reported with a moderate follow-up rate of 60–79%.

**Table 2:** Quality assessment of included studies on effectiveness of activity-based interventions based on the EPHPP tool

Study design	Study (year)	Selection bias	Study design	Confounders	Blinding	Data collection method	Withdrawals and drop-outs	Global rating
RCT	DiNapoli et al. [36] (2016)	moderate	strong	strong	moderate	strong	moderate	**strong**
TS	Gitlin et al. [37] (2016)	moderate	moderate	weak	weak	strong	weak	**weak**
	Weber et al. [38] (2009)	moderate	moderate	strong	weak	strong	weak	**weak**
BA	Cheong et al. [39] (2015)	moderate	moderate	weak	weak	weak	weak	**weak**
	Daykin et al. [40] (2017)	weak	weak	weak	weak	weak	weak	**weak**
PC	Windle et al. [41] (2018)	weak	moderate	strong	weak	weak	strong	**weak**

*BA* before-after study, *RCT* randomized controlled trial, *PC* prospective cohort study, *TS* time series

The remaining five studies received ‘weak’ ratings on two or more study quality components. More specifically, potential high risk for selection bias was present in two studies [40, 41] largely due to inadequate reporting around the target population or low participation rates. Three of the studies did not adequately describe the reliability or validity of the data collection tools [39–41]. Ratings for assessor and participant blinding indicated potential risk of detection and reporting bias: in three studies it was clear that assessors were aware of the intervention status of participants [37, 39, 40], and none of the studies described participant blinding. Control of confounders was limited or not described in half of the studies [37, 39, 40], however the remaining studies received strong ratings for that section. Four studies did not describe withdrawals and drop-outs or reported less than 60% follow-up rate (as per EPHPP tool components) [37–40]. Overall, the quality of the six included studies evaluating effectiveness in improving experience of care in hospital appears to be poor, mainly due to potential biases in terms of confounding, blinding, and short follow-up periods.

### Data synthesis

#### Intervention characteristics and components

Additional file 3*p*rovides a summary of intervention characteristics of included studies using the TIDieR [32] checklist items, and Additional file 4 provides a summary of intervention components. Despite including different activities or program structure, all six studies evaluating activity-based interventions were essentially driven by the idea that behavioral problems of Plwd represent unmet emotional or social needs, and cultural or social activities have the potential to address these needs by increasing engagement or re-engaging people in their environment and meaningful activities, promoting communication and connection to others, and improving wellbeing. All interventions were directly received by Plwd. Support and an inclusive approach to carers was part of the intervention in four studies [37, 38, 40, 41] while one relied on information provided by carers to formulate strategies and plan the intervention [37]. Studies evaluated either single or multiple activities usually performed in groups including music [36, 38–40], art [36, 41], reminiscence, board games [36], or movement therapy and sociotherapy in the context of a psychodynamic therapeutic community program [38]. Playing card games, crocheting, folding towels or seating exercises were part of the ‘activity prescriptions’ developed for the Tailored Activity Program for Hospitalized patients with behavioral symptoms (TAP-H [37]). Comparators included ‘usual’ care with the control group receiving some of the components received by the intervention group [36], or active comparators such as standardized activity sessions (instead of tailored activities [37]), and an unstructured social activity (without involving art activity [41]). The remaining studies used pre-intervention estimates as the comparator [38–40].

Interventions were delivered by a range of providers including artists and music/occupational/ recreation therapists, nursing staff, physicians, and social workers. Training to prepare providers to deliver the intervention was reported in two studies [37, 41]. The duration of interventions varied depending on the type of activity and program within the hospital facility, ranging from 30 min/day or 2 h/week for music therapy [39, 40] to 6-h programs for 2–3 times/week [38]. The location of activity-based interventions was usually described based on the type of facility or ward (e.g. acute care, psychiatric day hospital) except for one study [40] which specified that the music activity took place in an activity room close to the ward. Five of the included studies described tailoring activities or strategies to manage responsive behaviors (the preferred term, rather than challenging behaviors for actions, words and gestures that are a response to Plwd personal, physical or social environment) or provide person-centered care by taking into account patient needs, preferences, capabilities, and degree of cognitive impairment [36–39, 41]. Finally, modifications during early stages of the study were reported in Windle et al. [41]. Recruiting from a day care service for Plwd in addition to NHS assessment units after the second wave of intervention delivery was a protocol modification for that intervention [41]. Two studies [36, 41] reported on strategies to assess or improve fidelity and presented a varying degree of detail on the extent of intervention fidelity upon study completion.

#### Effectiveness of activity-based interventions

Included studies measured a number of outcomes for Plwd that can be collectively described as ‘aspects of wellbeing’ including: quality of life [36, 41], wellbeing [41], patient engagement [37–39, 41], and mood/emotional state [37, 39, 40]. Differences in study design or insufficient data to calculate effect sizes meant that studies under the same outcome category could not be meta-analyzed. However, effects sizes, confidence intervals, and/or *p*-values were calculated to aid interpretation of findings where sufficient data were available.

##### Patient engagement

Table 3 shows means and SD for control/pre-intervention and intervention/post-intervention groups for each aspect of wellbeing outcome (where these were reported or could be estimated). Four studies [37–39, 41] provided evidence regarding the effect of activity-based interventions on patient engagement (Table 3). In the TAP-H intervention [37], patients showed increased positive gestures but decreased positive statements compared to baseline behaviors. A decrease in negative statements and nonverbal behaviors was also observed (e.g. repetitive statements, verbal aggression, motoric or facial disturbances). Increased constructive and passive engagement (e.g. motor or verbal behaviors in response to the activity), and decreased self- or non-engagement (e.g. purposeless behavior involving engagement with self, staring into space) was also observed during music sessions compared to sessions without music in the study evaluating a creative music therapy intervention [39]. A third study assessing the impact of a psychotherapeutic day hospital program [38] using a time-series design found that the intervention was associated with better clinical progress in group therapy across the different time points (*β* = 2.01, *p* = 0.04). There was a lack of evidence for a positive impact of the visual arts program [41] on communication measured by a scale covering a range of behaviors related to engagement such as conversation, awareness, pleasure, humor and responsiveness. Patients’ communication actually deteriorated between baseline and the 3-month and 6-month time points, with study authors reporting significantly more difficulties in communication [41]. Calculated effect sizes also indicate a detrimental effect on communication (3 months: *d* = 0.64; CI: − 0.05 to 1.34, *p* = 0.07; 6 months: *d* = 0.76; CI: − 0.06 to 1.58, *p* = 0.06) but the wide confidence intervals suggest imprecision of the effect estimate possibly due to the low number of participants (baseline *n* = 19, 3-month time point *n* = 15, 6-month time point *n* = 9).

**Table 3:** Results for effectiveness of activity-based interventions to improve experience of care outcomes for Plwd in hospital

Study	Outcome (tool)	Comparison group (or pre-intervention)			Intervention group (or post-intervention)			Effect size *d* (95% CI)	Mean difference (95% CI)	p	Significant change
		N	Mean	SD	N	Mean	SD				
DiNapoli et al. [36] (2016)	Quality of life (DQoL)	13	3.27	0.72	21	3.64	0.66	0.53 (− 0.17 to 1.23)	0.37 (− 0.12 to 0.86)	0.13	↔
	BPSD (NRS-R)	26	10.04	6.97	26	7.19	5.58	−0.45 (− 0.99 to 0.11)	−2.85 (−6.37 to 0.67)	0.11	↔
Gitlin et al. [37] (2016)	Emotional state (pleasure-AARS)	10	9.9	range 2–20	15	13.76	range 0–57	NA	(increase)	NA	NA
	Mood (general alertness-AARS)	10	88.7	range 45–139	15	75.68	range 17–174	NA	(decrease)	NA	NA
	Mood (anxiety and anger-AARS)	10	22.6	range 0–34	15	5.3	range 0–29	NA	(decrease)	NA	NA
	Patient engagement (positive verbalisations)	10	56.2	range 15–126	15	40.67	range 0–116	NA	(decrease)	NA	NA
	Patient engagement (negative verbalisations)	10	17.5	range 0–84	15	5.58	range 0–32	NA	(decrease)	NA	NA
	Patient engagement (positive nonverbal)	10	38.6	range 9–65	15	41.87	range 1–152	NA	(increase)	NA	NA
	Patient engagement (negative nonverbal)	10	11.6	range 0–21	15	5.72	range 0–36	NA	(decrease)	NA	NA
Weber et al. [38] (2009)	Patient engagement (therapeutic progress-GES)	76	NA	NA	76	NA	NA	NA	(increase, β = 2.01)	0.04	↑
	Behaviour-Neuropsychiatric symptoms (NPI)	76	NA	NA	76	NA	NA	NA	(decrease, β = −4.21)	< 0.001	↑
Cheong et al. [39] (2016)	Mood (pleasure and general alertness-OERS)	25	0.68	NA	25	3.12	NA	NA	(increase)	0.01	↑
	Mood (anger, anxiety and sadness-OERS)	25	0.48	NA	25	0.32	NA	NA	(decrease)	0.05	↑
	Patient engagement (Constructive & passive-MPES)	25	6.26	NA	25	8.0	NA	NA	(increase)	0.01	↑
	Patient engagement (self- and non-engagement-MPES)	25	1.04	NA	25	0.72	NA	NA	(decrease)	0.01	↑
Daykin et al. [40] (2017)	Mood (happiness-ArtsObs)	20	NA	NA	20	NA	NA	NA	(increase)	NA	NA
	Patient engagement (relaxation, distraction, engagement-ArtsObs)	20	NA	NA	20	NA	NA	NA	(positive impact)	NA	NA
	Behaviour (agitation-ArtsObs)	20	NA	NA	20	NA	NA	NA	(decrease)	NA	NA
Windle et al. [41] (2018)	Quality of life (DEMQOL self-report, 3 months)	15	91.5	14	13	92.5	10.7	0.08 (−0.67 to 0.82)	1.0 (−8.80 to 10.80)	0.84	↔
	Quality of life (DEMQOL self-report, 6 months)	15	91.5	14	12	90.3	14.6	− 0.08 (− 0.84 to 0.68)	−1.20 (− 12.58 to 10.18)	0.83	↔
	Quality of life (DEMQOL proxy, 3 months)	19	86.7	12.6	9	96.3	10.2	0.78 (− 0.04 to 1.60)	9.60 (− 0.31 to 19.51)	0.06	↔
	Quality of life (DEMQOL proxy, 6 months)	19	86.7	12.6	4	85.5	15.6	−0.09 (−1.17 to 0.99)	− 1.20 (− 16.15 to 13.75)	0.87	↔
	Patient engagement (communication*, 3 months)	19	12.9	9.5	15	19.3	10	0.64 (−0.05 to 1.34)	6.40 (−0.44 to 13.24)	0.07	↔
	Patient engagement (communication*, 6 months)	19	12.9	9.5	9	20.7	10.9	0.76 (−0.06 to 1.58)	7.80 (−0.48 to 16.08)	0.06	↔
	Wellbeing domains (GCCWBOT, 2 weeks)										
	Interest	18	52.5	28.9	20	50.5	18.8	−0.08 (−0.72 to 0.56)	−2.0 (−17.88 to 13.88)	0.80	↔
	Attention	18	67.5	21	20	71.9	16.5	0.23 (−0.41 to 0.87)	4.4 (−7.96 to 16.76)	0.48	↔
	Pleasure	18	26	22.2	20	25.9	14.0	−0.01 (−0.64 to 0.63)	−0.10 (−12.18 to 11.98)	0.99	↔
	Normalcy	18	46.1	20.2	20	41.5	15.3	−0.25 (−0.89 to 0.39)	−4.60 (−16.32 to 7.12)	0.43	↔
	Self-esteem	18	29.2	5.5	20	27.9	6.4	−0.21 (−0.85 to 0.43)	−1.30 (−5.25 to 2.65)	0.51	↔
	Disengagement	18	19.8	25.0	20	19.0	24.0	−0.03 (−0.67 to 0.60)	−0.80 (−16.93 to 15.33)	0.92	↔
	Sadness	18	2.1	6.4	20	2.1	7.0	0.0 (−0.64 to 0.64)	0.0 (−4.43 to 4.43)	1.00	↔
	Negative affect	18	2.8	5.0	20	1.3	2.9	−0.36 (−1.01 to 0.28)	−1.50 (−4.16 to 1.16)	0.26	↔
	Wellbeing domains (GCCWBOT, 3 months)										
	Interest	18	52.5	28.9	12	47.9	18.3	−0.18 (−0.91 to 0.55)	−4.6 (−23.89 to 14.69)	0.63	↔
	Attention	18	67.5	21	12	69.5	20.4	0.09 (−0.64 to 0.82)	2.00 (−13.85 to 17.85)	0.80	↔
	Pleasure	18	26	22.2	12	25.5	18.9	−0.02 (−0.75 to 0.71)	−0.50 (−16.51 to 15.51)	0.95	↔
	Normalcy	18	46.1	20.2	12	44.3	13.8	−0.10 (−0.83 to 0.63)	−1.80 (−15.51 to 11.91)	0.79	↔
	Self-esteem	18	29.2	5.5	12	30	5.3	0.14 (−0.59 to 0.87)	0.80 (−3.34 to 4.94)	0.70	↔
	Disengagement	18	19.8	25.0	12	15.9	16	−0.17 (−0.90 to 0.56)	−3.90 (−20.63 to 12.83)	0.64	↔
	Sadness	18	2.1	6.4	12	0.4	1.3	−0.33 (−1.06 to 0.41)	−1.70 (−5.56 to 2.16)	0.37	↔
	Negative affect	18	2.8	5.0	12	1.7	2.6	−0.25 (−0.99 to 0.48)	−1.10 (−4.32 to 2.12)	0.49	↔

*AARS* Apparent Affect Rating Scale, *BPSD* Behavioural and psychological symptoms of dementia, *CI* Confidence interval, *d*: Cohen’s *d*, *DEMQOL* Dementia Quality of Life, *DQoL* Dementia Quality of Life instrument, *GCCWBOT* Greater Cincinnati Chapter Well-Being Observation Tool, *GES* Group Evaluation Scale, *MPES* Menorah Park Engagement Scale, *NRS-R* Neurobehavioral Rating Scale-Revised, *NA* Indicates relevant data was not reported or calculable, *OERS* The Lawton Observed Emotion Rating Scale, *SD* Standard deviation, *p*-value: for the mean difference between groups/pre-post

*: higher scores indicating more communication difficulties, ↑: statistically significant difference for outcome in this comparison and direction of effect beneficial for intervention group or post-test, ↓: statistically significant difference for outcome in this comparison and direction of effect not beneficial for intervention group or post-test, ↔: no statistically significant difference for outcome in this comparison

##### Mood

Mood and emotional states of Plwd were assessed in three studies. The authors of the creative music therapy study [39] reported significantly higher frequency of positive mood ratings (general alertness and pleasure; *p* = 0.01) and lower observations of negative mood states (anxiety, anger, sadness; *p* = 0.04) during music sessions compared to sessions without music. However, only a small number of participants were observed (*n* = 25) and the study did not provide additional details about reasons for admission or other conditions the patients may had been exposed to while on the unit. Gitlin et al. [37] assessed the same emotional states in 15 Plwd comparing observations during a baseline standardized activity to the TAP-H intervention sessions. There were insufficient data to calculate effect sizes as authors only compared average percentage of time participants engaged in behaviors: patients showed increased pleasure, and decreased alertness and negative mood states in intervention sessions compared to baseline. In the third study [40], observational data indicated that participants’ happiness scores increased by the end of each participatory music-making session, and the impact on engagement, distraction and relaxation was also consistently positive (statistical comparisons not reported).

##### Wellbeing and quality of life

The impact of the visual arts program [41] was assessed on eight domains of wellbeing using an observation tool compared to an unstructured social activity not involving art. The study was conducted across three settings one of which was NHS hospital wards providing care to 23 Plwd. Although some of the assessed domain scores improved at the 2-week and 3-month time points compared to the baseline activity scores (e.g. attention, sadness, disengagement, negative affect), overall there was a lack of evidence to support a beneficial effect of the program on wellbeing. Quality of life was also assessed in the same program [41] along with a second study trialing the effectiveness of individualized social activities [36] in older people with cognitive impairment. Neither of the interventions were found to be effective at improving proxy- [41] or self-reported [36, 41] quality of life. The wide confidence intervals suggest imprecision of the effect estimates possible due to the small sample size (see Table 3).

##### Behavior

Behavioral outcomes were assessed in three studies (see Table 3). The RCT examining the effectiveness of individualized social activities [36] showed lower scores on a scale measuring BPSD but there was no significant difference between groups post-intervention (*d* = − 0.45; 95% CI: − 0.99 to 0.11, *p* = 0.11). However, a psychotherapeutic day hospital program [38] including music, movement, psychodynamic and sociotherapy was associated with a statistically significant reduction in neuropsychiatric symptoms across time points from admission to discharge (linear regression β = − 4.21, *p* < 0.001), particularly anxiety and apathy. Observational data at the start and the end of the participatory music-making intervention [40] indicated consistently positive effects with reduced agitation of participants, although authors did not provide additional comparative data.

Overall, there were positive trends regarding evidence for the effectiveness of activity-based interventions to improve experience of care for Plwd as reflected by aspects of wellbeing measures during their stay in hospital settings.

## Discussion

This review synthesized the evidence on the effectiveness of activity-based interventions to improve the experience of care for Plwd in hospital. Findings indicate potential beneficial effects regarding effectiveness on Plwd engagement, mood and behavior although studies were underpowered and of low methodological quality. Although lack of strong evidence does not equal lack of effectiveness, it limits the conclusions that can be drawn based on the available published studies.

Literature on models of care for Plwd emphasizes the benefits that come with care environments that meet the physical, social and emotional needs of Plwd [42, 43]. All included studies were driven by the principle that activity-based interventions can improve wellbeing by addressing unmet needs of Plwd. According to the need-driven dementia-compromised behavior model [44], responsive behaviors reflect needs that Plwd are unable to articulate clearly. Need-driven behaviors result from the interaction of salient background factors (e.g. neurological, cognitive, health status and psychosocial factors) and more changeable proximal factors (psychological and physiological needs, and aspects of the physical and social environment). Seen this way, these behaviors become meaningful and enable carers to identify possible approaches to intervention. Indeed, sensory deprivation and lack of meaningful engagement in a potentially overwhelming hospital environment may affect wellbeing and responsive behaviors of Plwd. Therefore, intervention strategies that consider and target identified need states of Plwd in hospital are likely to improve experience of care. While person-centered and need-driven approaches promoting quality of care and wellbeing have gradually been integrated into long-term care settings, they do not seem to be standard practice within acute care settings.

Previous research around experience of care for Plwd has either focused on particular set of outcomes or has not been specific to hospital settings. Oliveira et al. [27] reviewed 20 studies of non-pharmacological interventions in the community or residential care settings to reduce BPSD concluding that activity programs (e.g. music therapy, art activities, physical exercise) were the most common type of intervention with agitation as the most responsive symptom. Activity interventions were also among those reported to be effective in improving mood and quality of life in more recent reviews of non-pharmacological interventions for Plwd living at home or in care homes [23, 24, 45]. However, none of the reviews included hospital-based interventions, or could allude to the generalizability of these findings to acute care settings as a number of factors including reasons for admission and treatment provided to Plwd may influence mood and BPSD during hospitalization. By focusing our question on experience of care in hospitals, we were able to capture relevant studies to draw a picture of the evaluated activity-based interventions across a range of outcomes (including behavioral indicators although only as a secondary outcome) within a number of hospital settings including acute care and psychogeriatric units. Our review is in line with the direction of findings in previous research conducted in the community and care homes, and provides preliminary yet not conclusive evidence for the effectiveness of activity-based interventions to improve experience of care for Plwd in hospital.

### Strengths and limitations

This is the first systematic review of studies evaluating the effectiveness of activity-based interventions to improve the experience of care for Plwd while in hospital. We performed comprehensive search strategies to identify published research including major electronic databases, backward and forward citation searching. There are however a number of limitations predominantly related to the primary studies included in the review. We identified a small number of studies meeting the inclusion criteria which were often underpowered. While this is likely a reflection of the methodological challenges of conducting hospital-based research in dementia care, it also means that conclusions about the effectiveness of activity-based interventions on experience of care are drawn based on a limited body of evidence. Although all interventions were centered around activities targeting potential unmet needs, intervention components and the specific activities varied across studies; along with the aforementioned limitations, this means that there is no robust evidence for specific types of activities. It is possible that relevant interventions may have been excluded as studies reporting behavioral outcomes for Plwd were not included unless they also reported other experience of care indicators. Reason for admission was not reported in all studies and it is likely that Plwd may have received pharmacological and/or other treatments during their hospitalization for acute or existing comorbidities. Dementia can magnify difficulties in experience of care in people with comorbid conditions and complicate the delivery of care in hospital. The presence of dementia can often dominate clinical encounters possibly subjecting Plwd with comorbidities to delayed recognition or management of symptoms. It is also possible that the severity of comorbidities and overall health status of Plwd may have influenced (level of) participation to the identified activity-based interventions. These factors were not accounted for in the studies and may have affected the outcomes. For example, Plwd are at increased risk of delirium (common in hospitalized older patients), and we cannot exclude the potential role that the presence of unreported delirium may have played on the results. There was an insufficient number of studies with compatible characteristics to allow meta-analyses of different outcomes. However, we calculated SMDs to aid interpretation of findings.

### Implications for practice

The RCN SPACE principles [13] for improving dementia care in hospital settings highlight the importance of skilled staff who have time to care, partnership working with carers, assessment and early identification, care that is individualized, and environments that are dementia friendly. Although primarily intended for professional care providers who work with Plwd and their families in residential and community-based care settings, the Alzheimer’s Association dementia care practice recommendations also reflect very similar principles in the areas of person-centered care, ongoing care for BPSD, and support for activities of daily living, staffing, and supportive and therapeutic environments [19]. Identified activity interventions included components that can support these care principles. For example, two of the included studies trained care staff in the activities of the interventions, and four studies reported the addition of specialist capacity (e.g. social worker, certified music therapist) as part of the intervention. Additionally, all of the studies attempted to individualize the care provided either by asking Plwd about their preferences or gathering life story information and details of the interests of Plwd from their family members. By definition, all studies also provided either space or resources to support activity and stimulation. Although our findings do not provide definitive evidence for the effectiveness of the described activity interventions, the studies did not report harmful effects. In the one study reporting deterioration in communication [41], the authors attributed this change to the health status and potentially more intensive care needs of participants in the assessment units compared to those in the residential care setting also examined in the same study. However, studies indicated that activity interventions in hospital are feasible with the potential to improve aspects of experience of care for Plwd. Therefore, our findings align with the SPACE principles and Alzheimer’s Association recommendations and encourage further implementation of activity interventions in hospital settings to strengthen the evidence for the best way to improve patient experience and quality of dementia care in hospitals.

### Recommendations for research

Existing studies point towards the need for methodological improvements in this area. In light of limited resources, it is important that the evidence base is informed by larger well-conducted controlled studies of interventions that are easy to implement. Most of the included studies showed positive trends in improving experience of care outcomes, and provided activities tailored to patient preferences and abilities. This suggests activity interventions are beyond a ‘one size fits all’ approach, and that individualization of activities may be a key component of their effectiveness. Improved reporting of methods and intervention details is therefore essential to facilitate comparisons across different interventions. The numerous dimensions of experience of care makes it difficult to measure, but consensus over standardized outcome measures would also facilitate comparisons and pooling of data across studies. The addition of process and economic evaluations to studies evaluating effectiveness would also help to understand why some activity interventions may or may not work, and make links with the performance-based measures that often drive change in hospitals. Information from qualitative studies can also be used to inform intervention effectiveness by helping to create hypotheses about why an intervention did or did not work, and as such can facilitate implementation of interventions in other settings. For instance, one of the included studies [40] included qualitative data and revealed additional information about patients arriving at music sessions in various moods, some appearing not to know where they were or why they were there, but becoming more engaged as the group progressed. Some participants enjoyed the social aspect of the music sessions or encouraged other patients to sing when they got back to the ward. Observations also showed the music project seemed to have a positive effect on staff and the clinical environment, while sometimes staff organized their shift to fit around sessions, striving to protect the time allocated to music on the ward. Therefore, using both quantitative and qualitative methods to assess experience of care is vital in ascertaining evidence on how to improve hospital dementia care, and staff-related outcomes may prove to be alternative informative measures of intervention effectiveness relating to experience of care. The effectiveness of interventions in different hospital settings also warrants further exploration. Admissions to acute care settings are more likely to be due to physical health problems whereas the reason for admission to psychogeriatric wards is likely to be linked to behavioral issues, and these factors may also affect experience of care and intervention effectiveness.

## Conclusions

The small number of identified studies indicate that activity-based interventions implemented in hospitals may be effective in improving aspects of experience of care for Plwd including patient engagement, their mood and other behavioral indicators. Larger well-conducted studies are needed to fully evaluate the potential of activity interventions to improve experience of care in hospital settings, and the extent to which they may also benefit staff wellbeing and the wider ward environment.

## Supplementary information

**Additional file 1.** Appendix 1: MEDLINE search strategy

**Additional file 2 Table S1**. Outcome categories and measures within studies assessing effectiveness of activity interventions for Plwd

**Additional file 3 Table S2**. Summary of intervention details of included studies using TIDieR items

**Additional file 4 Table S3**. Intervention components across included studies for the effectiveness of activity-based interventions to improve experience of care in hospital for Plwd

## Data Availability

The reviewed studies supporting the findings and on which the conclusions of the manuscript rely can be found in the reference list.

## Abbreviations

BPSD: Behavioral and psychological symptoms of dementia
CI: Confidence interval
NHS: National health service
Plwd: Person or people living with dementia
PRISMA: Preferred reporting items for systematic reviews and meta-analyses
RCN: Royal college of nursing
RCT: Randomized controlled trial
SD: Standard deviation
SMD: Standardised mean difference
SPACE: Staff, partnership, assessment, individualized care and environments
TAP-H: Tailored activity program for hospitalized patients with behavioral symptoms
TIDieR: Template for intervention description and replication checklist
