# Supplementary material for: Activity interventions to improve the experience of care in hospital for people living with dementia: a systematic review
Source: BMC Geriatr. 2020 Apr 10;20:131. doi: 10.1186/s12877-020-01534-7 (PMC7146899; doi:10.1186/s12877-020-01534-7)
Supplement: Supplementary file 1 — Additional file 1. Appendix 1: MEDLINE search strategy [file 12877_2020_1534_MOESM1_ESM.pdf]

**Additional file 1**

**Appendix 1** - MEDLINE search strategy

Database: Ovid MEDLINE(R) Epub Ahead of Print, In-Process & Other Non-Indexed Citations, Ovid MEDLINE(R) Daily and Ovid MEDLINE(R) <1946 to Present>

Search Strategy:

--------------------------------------------------------------------------------

1 exp Dementia/nu, px, rh, th [Nursing, Psychology, Rehabilitation, Therapy]

2 exp Delirium/nu [Nursing]

3 exp Confusion/nu [Nursing]

4 dementia.ti,ab.

5 alzheimer*.ti,ab.

6 (cognitive adj2 (disorder* or dysfunction or impair*)).ti,ab.

7 delirium.ti,ab.

8 or/1-7

9 Hospitals, General/ma, mt, og, st, ut [Manpower, Methods, Organization & Administration, Standards, Utilization]

10 general hospital*.ti,ab.

11 acute hospital*.ti,ab.

12 rural hospital*.ti,ab.

13 (emergency department* adj5 (dementia or alzheimer*)).ti,ab.

14 (acute adj2 care).ti,ab.

15 (hospital* adj2 (care or setting*)).ti,ab.

16 (general adj3 ward*).ti,ab.

17 (acute adj3 ward*).ti,ab.

18 (acute adj3 setting*).ti,ab.

19 (admission adj3 hospital*).ti,ab.

20 ((ambulance or paramedic) adj5 care).ti,ab.

21 (discharge adj2 hospital).ti,ab.

22 or/9-21

23 Patient Care Management/

24 (patient centered or patient centred).ti,ab.

25 (person centered or person centred).ti,ab.

26 (personal adj (care or hygiene)).ti,ab.

27 (dignity adj2 care).ti,ab.

28 dementia friendly.ti,ab.

29 hairdressing.ti,ab.

30 rummage box*.ti,ab.

31 reminiscence.ti,ab.

32 artful moments.ti,ab.

33 activities.ti,ab.

34 (training adj10 (nurse* or doctor* or staff or health professionals or healthcare professionals or healthcare assistants or cleaners or porters or receptionists)).ti,ab.

35 (education* adj10 (nurse* or doctor* or staff or health professionals or healthcare professionals or healthcare assistants or cleaners or porters or receptionists)).ti,ab.

36 workshop*.ti,ab.

37 (dementia adj3 specialist*).ti,ab.

38 (liaison adj (worker* or staff or nurse*)).ti,ab.

39 ((patient or person) adj liaison).ti,ab.

40 ("one to one" adj (care or monitoring)).ti,ab.

41 champion.ti,ab.

42 (individual adj (care or monitoring)).ti,ab.

43 constant* monitor*.ti,ab.

44 T A DA method.ti,ab.

45 segregat*.ti,ab.

46 befriend*.ti,ab.

47 (visitor* adj5 (hospital* or ward*)).ti,ab.

48 (volunteer* adj5 (hospital* or ward*)).ti,ab.

49 (dementia adj2 friend*).ti,ab.

50 AMIGOS.ti,ab.

51 (ward adj3 (design or ambience or decor*)).ti,ab.

52 ((hospital or ward) adj environment).ti,ab.

53 (dementia adj2 ward*).ti,ab.

54 (speciali*ed adj2 (ward* or unit*)).ti,ab.

55 dementia pods.ti,ab.

56 pods programme*.ti,ab.

57 (garden* or outdoor* or outside or window or sunlight or daylight or flower* or nature).ti,ab.

58 ((ward or hospital or organisation*) adj2 culture).ti,ab.

59 ward life rhythm.ti,ab.

60 ambience.ti,ab.

61 lighting.ti,ab.

62 mealtime*.ti,ab.

63 (medication adj (routine* or regime* or process*)).ti,ab.

64 ("end of life" adj5 care).ti,ab.

65 ((advanced or palliative) adj care).ti,ab.

66 or/23-65

67 8 and 22 and 66
