# Supplementary material for: Activity interventions to improve the experience of care in hospital for people living with dementia: a systematic review
Source: BMC Geriatr. 2020 Apr 10;20:131. doi: 10.1186/s12877-020-01534-7 (PMC7146899; doi:10.1186/s12877-020-01534-7)
Supplement: Supplementary file 2 — Additional file 2 Table S1. Outcome categories and measures within studies assessing effectiveness of activity interventions for Plwd [file 12877_2020_1534_MOESM2_ESM.pdf]

| Additional file 2- Table S1. Outcome categories and measures within studies assessing effectiveness of activity interventions for Plwd | | | |
| --- | --- | --- | --- |
| Study,  design | **Outcome category** | **Outcome measure** | **Definition/scale items if reported or identified** |
| DiNapoli et al. (2016)  RCT | Quality of life | Dementia Quality of Life instrument (DQoL) | The Dementia Quality of Life instrument is 29-item direct-interview instrument used to assess QoL in mild to moderate dementia participants. It has two distinct factors: negative affect (11 items) and General DQoL (comprising the remaining 18 items). To assess QOL, this study used an average score of the items that comprise the General DQoL (a=0.81). A global item of QOL (i.e., ‘Overall, how would you rate your quality of life?’) was also used to determine participant inclusion. The General DQoL and global item scores range from 1 (never or bad) to 5 (very often or excellent), with higher scores indicating greater QoL |
|  | Behaviour  (cognitive, psychiatric, and behavioural symptoms; BPSD) | Neurobehavioral Rating Scale-Revised | The NRS-R is a 29-item direct-interview instrument and each item is scored on a scale of 0 (Absent) to 3 (Severe). An overall score was calculated by summing the responses, with higher scores indicating greater presence of behavioural and psychological symptoms (a= 0.83). The NRS-R has been shown to be a reliable assessment measure of BPSD. |
| Gitlin et al.  (2016)  TS | Patient engagement | 1.following treatment sessions, OTs completed 10 questions about patients  2.Direct observation to evaluate behavioural and affective reactions | First, following treatment sessions, OTs completed 10 questions (e.g., patient “appears bored or disinterested,” “shows signs of interest,” “expresses or shows feelings of pleasure,” “conveys that she/he wanted to stop”). Each item was scored from not at all (0) to extremely (4) for a possible range of 0–40. Higher scores indicated greater engagement.  Second, direct observation was used to evaluate patient behavioural and affective reactions. Video recordings were conducted in a private room on the hospital unit designated for the study. To establish a baseline, each enrolled patient was observed within 3 days of admission and prior to OT prescribed activities, using a standard activity such as CNAs providing patients with a magazine for up to 15 min. Then, patients were observed in up to six TAP-H sessions involving the introduction or use of activities (Sessions 3–8) over 3 weeks or time-to-discharge.  Video-recordings were subsequently coded and analysed across three behavioural domains (affect, verbal, and nonverbal behaviours) by up to five raters. Codes for each behavioural domain were identified from previously developed protocols. Affect codes were based upon the Apparent Affect Rating Scale and involved two positive emotional states (general alertness and pleasure) and three negative emotional states (anxiety, anger, and sadness). Verbal and nonverbal behaviour codes were also based upon protocols from previous observational studies with this population. Verbal behaviours were coded into 11 categories: positive, repetitive, nonsensical, aggressive, delusions, expressions of abandonment, somatic, sexual, self-harm, rejection of care or activity, and depressive. Nonverbal behaviours involved 12 categories: positive gestures, positive touch, motoric disturbance, facial disturbance, aggression, eyes closed, somatic, self-harm, sexual, rejection of care or activity, distracted, or depressive behaviours. Behaviours not observed were coded as “Can’t see.” |
| Weber et al.  (2009)  TS | Patient engagement  (therapeutic progress of group) | 17-item tool (GES) | The Group Evaluation Scale (GES) was used to assess group progress in the elderly. It is rated on a 5-point scale that evaluates satisfaction, perception of group’s purpose, relevance, affective climate, cohesion, quality of interaction, personal involvement and adhesion to group treatment |
|  | Behaviour  (neuropsychiatric symptoms) | The Neuropsychiatric Inventory (NPI) | The NPI assesses 12 neuropsychiatric symptoms commonly observed in dementia: delusions, hallucinations, agitation, depression, anxiety, euphoria, apathy, disinhibition, irritability, aberrant motor behaviour, night-time behaviour disturbances, as well as appetite and eating disorders. The severity (1–3) and frequency (1–4) of each symptom are rated in an interview with the caregiver on the basis of scripted questions, generating a composite score for each of the 12 domains (product of frequency and severity subscores, range 0–12) as well as a total NPI score (sum of all composite scores for each domain 0–144).  Content and concurrent validity, as well as inter-rater and test–retest reliability are established, and the NPI has proven sensitive to treatment efficacy. |
| Cheong et al. (2016)  BA | Patient engagement | Menorah Park Engagement Scale | The Menorah Park Engagement Scale was used to assess engagement. It captures four types of engagement: constructive engagement, passive engagement, self-engagement, and non-engagement.  Constructive engagement referred to any motor or verbal behaviour exhibited in response to the activity the patient was participating in. Passive engagement pertained to listening and/or observing in response to the activity presented.  Self-engagement was defined as any purposeless behaviour involving the patient’s engagement with himself or herself during the activity. Non-engagement related to staring into space or another direction away from the activity for >10 s or sleeping during an activity. The duration of each type of engagement was recorded in 5-min periods |
|  | Emotional state/mood | Observed Emotion Rating Scale | The Lawton Observed Emotion Rating Scale was used to measure affect. The five types of emotion recorded included: pleasure, general alertness, anger, anxiety/fear, and sadness. Observers used standardized guidelines on facial and vocal expressions to code the different categories of emotion. |
| Daykin et al. (2017)  BA | Emotional state/mood | Arts Observational Scale (ArtsObs) | Observational data were collected using ArtsObs. This is a structured assessment tool that allows observers to record the impact of activities on participants’ mood, distraction and relaxation. These impacts are scored on a scale of 1 (no benefit or negative impact) to 3 (very positive impacts for individuals and the ward atmosphere). The tool also allows recording of individual participants’ happiness scores at the start and after the activity on a scale of 0 (negative, angry response) through to 7 (happy and excited) |
|  | Behaviour (agitation) | Arts Observational Scale (ArtsObs) | Observational data were collected using the Arts Observational Scale (ArtsObs). This is a structured assessment tool that allows observers to record the impact of activities on participants’ mood, distraction and relaxation. These impacts are scored on a scale of 1 (no benefit or negative impact) to 3 (very positive impacts for individuals and the ward atmosphere). The tool also allows recording of individual participants’ happiness scores at the start and after the activity on a scale of 0 (negative, angry response) through to 7 (happy and excited) |
| Windle et al. (2018)  PC | Quality of life  (self-report, proxy) | Dementia Quality of Life (DEMQOL) | The DEMQOL assesses five domains of QoL, including positive and negative emotions, memory, loneliness, and daily activities. It uses both self-reporting (29-items) and ratings by family carer or staff member as proxy (DEMQOL-Proxy; 32- items). Higher scores indicate better QoL. Respondents are asked to indicate how much they experienced each item during the last week (“a lot”=1; “quite a bit”=2; “a little”=3; “not at all”=4) |
|  | Patient engagement  (communication and social behaviour) | Communication Scale (HCS) (range 0-48); | Communication and social behaviour was assessed with the Holden Communication Scale completed by formal or informal carers. The scale consists of 12-items (range 0–48) covering a range of social behaviour and communication variables, including conversation, awareness, pleasure, humour, and responsiveness. Higher scores indicate *more difficulty* in communication |
|  | Wellbeing | Greater Cincinnati Chapter Well-Being Observation Tool (GCCWBOT) | The Greater Cincinnati Chapter Well-Being Observation Tool (GCCWBOT) was developed specifically to observe the effects of a visual arts activity on the well-being of people with dementia. The original instrument addresses seven domains of well-being with different numbers of indicators (interest, sustained attention, pleasure, negative affect, sadness, self-esteem, and normalcy). In this study, we the tool is used with five further indicators included: “interest in own work” (interest domain), “negative comments” (self-esteem domain), with an additional domain to capture disengagement represented by “neutral passivity,” “staring into space,” and “sleeping behaviour” |
|  | Behaviour  (BPSD) | The Neuropsychiatric Inventory (NPI) | No description in paper. See above Weber-2009 for description of NPI. |
| BA: before-after study, RCT: randomised controlled trial, PC: prospective cohort study, TS: time series | | | |
