# Supplementary material for: Activity interventions to improve the experience of care in hospital for people living with dementia: a systematic review
Source: BMC Geriatr. 2020 Apr 10;20:131. doi: 10.1186/s12877-020-01534-7 (PMC7146899; doi:10.1186/s12877-020-01534-7)
Supplement: Supplementary file 3 — Additional file 3 Table S2. Summary of intervention details of included studies using TIDieR items [file 12877_2020_1534_MOESM3_ESM.pdf]

| Additional file 3-Table S2. Summary of intervention details of included studies using TIDieR items* | | | | | | | | | | | | | |
| --- | --- | --- | --- | --- | --- | --- | --- | --- | --- | --- | --- | --- | --- |
| Study | **Brief name** | **Recipient** | **Why (rationale, theory or goals)** | **What (materials)** | **What (procedures)** | **Who provided** | **How (mode of delivery; individual/**  **group)** | **Where** | **When and how much** | **Tailoring** | **Modification of IV throughout study** | **Strategies to improve or maintain IV fidelity** | **Extent of IV fidelity** |
| DiNapoli et al. (2016) | Individualized Social Activities Intervention (ISAI) | Older adults with  mild to moderate cognitive impairment | The need-driven dementia-compromised behaviour model suggests that behavioural symptoms are an indication of unmet social needs. Interventions that increase participant’s engagement in meaningful social activities will likely improve QoL and decrease BPSD | Personal interests and functional  status assessed with the Assessment Tool for  Individualizing Activities. A fill-in-the-blank Participant Review gathered information  about participant’s family, religion, occupation, hobbies, music, and other personal interests | Therapists  matched the data from the assessments with participant  characteristics to prescribe a list of potential activities that are not routinely available within the facility | Trained research assistant | Not specified;  The most frequently delivered activities in the study  included reminiscence/life review or casual conversation,  puzzles/cards/board games, listening to music, and doing  art | State-supported geriatric psychiatric facility | IV group received the ISAI for approx. 30-60 minutes for up to 15 consecutive days | The intervention was  individualized based on participants’ interests, cognition,  and functional status | Not described | A treatment implementation model measuring individual treatment components was applied. Patient’s adherence with treatment regimen was assessed by the number of completed sessions, total time spent engaging in activities and participant engagement as perceived by therapist. Delivery was rated by an independent reviewer listening to one randomly chosen audiotape session from early and one from late in treatment. To learn structured interview assessment,  assessors practiced the assessment with the principal investigator  (PI) until an adequate mastery was obtained. All  assessments were audiotaped and the PI randomly reviewed about 10% of the recordings to ensure that the  delivery was standardized and appropriate | 61.5% participants engaged  in all 15 sessions of the ISAI; the average total time participants spent engaging  in activities was 616.88 minutes; therapists perceived the  participants as actively engaging in the activities;  98% of the brief quizzes  assessing participant’s understanding of the completed  activities were answered correctly; 93.1% of the sessions and 98.9% of the assessments  were appropriately delivered |
| Gitlin et al. (2016) | Tailored Activity Program for Hospitals  (TAP-H) | Plwd, family caregivers | Activities tailored to interests and abilities of Plwd may be useful by aligning cognitive/functional capacity with environmental expectations and enable individuals to positively re-engage in their environments. To understand its implementation potential and if TAP-H can be “normalized” within hospital settings, the study drew upon Normalization Process Theory | Not described | A research team member conducted brief telephone interviews with family members to obtain patient background information, and functional and behavioural profiles. TAP-H involves up to 11 sessions during hospitalization with three phases: assessment and activity planning, activity implementation and staff training (CNAs, RTs), and generalization (helping staff use strategies for care challenges; reviewing activities with families at discharge) | Occupational therapists (OTs), then Certified Nursing Assistants (CNAs) and Recreational therapists (RTs) | Brief telephone interview with families, face-to face with families and patients, discussion and coaching CNAs and RTs | Medical Behavioural  Unit | TAP-H involves up to 11 sessions during hospitalization that unfolds in three phases: assessment (sessions 1-2), implementation (sessions 3-7), generalization (sessions 8-10) | OTs interviewed families to obtain information about patient previous roles, habits, interests. Separate patient assessments informed activity choice, grading of activities to capabilities and strategies needed to support engagement | Not described | OTs documented number (dose) and duration (intensity) of sessions. Time in minutes was examined for each session  across participants and overall. CNAs and RTs also  recorded each time they used prescribed activities independently  of OT sessions | Average number of treatment sessions per patient was 8.00 (SD = 2.71, range = 3–13). Average time spent per session was 38.18 min (SD = 10.01, range = 19.09 –57.50).  CNAs and RTs used activities independently of OT sessions for 65 times (24 times = CNAs; 41 times = RTs) |
| Weber et al. (2009) | Psychotherapeutic day hospital program | Plwd and concomitant BPSD | Psychotherapeutic  interventions focusing on caregiver burden are thought to represent a useful complement to pharmacotherapy, and BPSD may be amended with person-centred psycho-dynamic interventions that address the emotional  needs of older Plwd | Not described | The intervention consists of music therapy, movement therapy, psychodynamic therapy and sociotherapy;  Family interventions include  the assessment of communication patterns, offer relief for caregivers’  burnout and opportunities to formulate a request for help; Liaison meetings  assure the coordination of professional caring networks  and provide continuous assessment and advice necessary for the success of a shared project | The care  team includes two residents in psychiatry, one senior  resident, one movement therapist, one music therapist,  one psychologist, one social worker and four nurses | Combination of individual and group psychotherapy. During each attended week, each patient participates in four mixed gender groups of maximum 10 participants. Participants’ personal goals and achievements, therapeutic  progress and drug management are discussed in individual interviews. The care program is completed by  a weekly administrative and organizational meeting  including both participants and staff members | Psychiatric day hospital | All participants attend the therapeutic community 2-3 times/week for a 6-hour day, including lunch | Not described | Not described | Not described | Not described |
| Cheong et al. (2016) | Creative music therapy (CMT) | Plwd/ delirium | Music therapy has been used to improve engagement and decrease agitation in Plwd mostly in long-term care settings, and it may hold promise in acute care settings. Main approach used was the Nordoff- Robbins CMT, a patient-centred, improvisational approach to individual and group music therapy based on the premise that every individual has an ability to respond to music | Not described | Plwd would have been receiving usual care before the music session, and would continue with usual care afterwards. The music therapist engaged the patient in both active music making or music listening and worked toward building a work of aesthetic value by embracing whatever musical material the patient offered | Board certified music therapist | Group or one-to-one music sessions | Acute care unit | Day 1: Plwd observed for 30 min without CMT at 3 times;  Day 2 & 3: Plwd observed for 30 min before and after a 30-min CMT session; study ran over a 3-month period | Based on individual’s profile and response to music,  the music therapist modified and adapted techniques to meet the patients’ needs; CMT sessions included playing familiar songs of patient’s choice | Not described | Not described | Not described |
| Daykin et al. (2017) | Inclusive participatory music activity to support wellbeing | Plwd | Rationale was to address the need for non-pharmacological approaches and evaluate their use in a busy hospital setting. Art therapies including music can help to reduce behavioural problems and promote communication and connection with others | Not described | Sessions lasting up to 2 hours were held in an activity room close to the ward. Following a similar structure, each session began with a brief performance of a classical piece on the viola by the musician, played as participants arrived and as tea and biscuits were being distributed. A series of participatory activities was next, beginning with singing familiar songs and playing hand-held percussion instruments. Reminiscence was one element, and activities also included song-writing and composing, and participants were invited to conduct using a baton as the musician improvised in response | Led by a professional  orchestral musician trained to work with Plwd | In groups of between five and eight,  participants (patients, their care staff, and visitors), attended sessions led by a professional  orchestral musician | Acute elderly care service in hospital, activity room | Weekly sessions lasting up to 2 hours for 10 weeks | Authors stated that intervention was not described as music therapy which is often individually based and focused on clinical goals | Not described | Not described | Authors stated that as some patients were  discharged during the study, each participant received on average 2-3 music sessions |
| Windle et al. (2018) | Visual arts program | Plwd | The program was developed for this research through a theoretical investigation of the contextual factors and mechanisms which shape outcomes. Cultural activities including  art interventions have the potential to improve a broad range of outcomes for Plwd including well-being, QoL, cognitive function, and communication | Training materials not described;  Materials provided depended on the task and included water-based paints,  pastels, colour pencils, collage material, glue, iPad,  quick drying modelling clay, and print-making  supplies | The community arts partners came together for training prior to the start of the study. Artists from each regional organization delivered the sessions in their respective geographical research site. Site 2 (NHS hospital wards) could not visit a gallery due to staff restrictions leaving the hospital, therefore the artists brought a small selection of artworks to the participants to facilitate discussions | Lead artist with prior experience and training in art and dementia facilitated each session, supported by a second artist | Groups sessions,  structured so that the first half was an art  viewing activity, focusing on a small number of artworks, followed by art-making | Across 3 research settings including one county hospital in Derbyshire | Each site  was expected to deliver four waves of the program,  each wave being 12 weeks in length, delivered once  a week for two hours to small groups (maximum of 15 participants) | Sessions were  flexible and dependent upon the varying degrees of  cognitive impairment presented to the artists | At site 2-hospital the protocol was modified after the second  wave of intervention delivery to also include  recruitment from a day care service for Plwd | Not described | A post-intervention review meeting with the artists indicated the program was delivered according to the core principles.  Across sites participants attended an average of 7 sessions |
| ^*^For an explanation and elaboration of each item, please refer to the TIDieR (Template for Intervention Description and Replication) checklist and guide. BMJ 2014;348:g1687 <http://www.bmj.com/content/348/bmj.g1687>;  BPSD: behavioural and psychological symptoms of dementia, CNAs: Certified Nursing Assistants, IV: intervention, Plwd: people with dementia, RTs: recreational therapists | | | | | | | | | | | | | |
