# Supplementary material for: Activity interventions to improve the experience of care in hospital for people living with dementia: a systematic review
Source: BMC Geriatr. 2020 Apr 10;20:131. doi: 10.1186/s12877-020-01534-7 (PMC7146899; doi:10.1186/s12877-020-01534-7)
Supplement: Supplementary file 4 — Additional file 4 Table S3. Intervention components across included studies for the effectiveness of activity-based interventions to improve experience of care in hospital for Plwd [file 12877_2020_1534_MOESM4_ESM.pdf]

| Additional file 4-Table S3. Intervention components across included studies for the effectiveness of activity-based interventions to improve experience of care in hospital for Plwd | | | | | | | | | | | | | | | |
| --- | --- | --- | --- | --- | --- | --- | --- | --- | --- | --- | --- | --- | --- | --- | --- |
|  | **Intervention components** | | | | | | | | | | | | |  | |
| Study  Author-  Date |  | **Therapeutic support to carers** | **Information/education for families** | **Inclusive approach to family carers** | **Activities for Plwd** | **Staff**  **training/education** | **Cementing new knowledge and practice through feedback on wards** |  | **Documentation for staff to improve individualised care** | **Approach adopted to guide caring for Plwd** | **Specialist capacity added** |  | **Total** | |  |
| DiNapoli et al. (2016) |  |  |  |  | ✓most frequently delivered: reminiscence/life review, casual conversation  puzzles/cards/board games, listening to music, doing  art |  |  |  | ✓Personal interests and functional  status was assessed with the Assessment Tool for  Individualizing Activities |  | ✓social worker to assist with discharge planning |  | 3 | |  |
| Gitlin et al. (2016) |  |  | ✓reviewing assessments & activities for home use |  | ✓ activity prescriptions (e.g. folding towels) | ✓ | ✓by OT |  | ✓ interview with family about patient roles, habits, interests  ✓patient assessment by OT |  |  |  | 6 | |  |
| Weber et al. (2009) |  | ✓assessment of communication patterns btw family members |  | ✓family interventions | ✓music therapy, movement therapy, psychodynamic  therapy and sociotherapy |  | ✓weekly team meetings |  |  | ✓Psychodynamic, therapeutic community |  |  | 5 | |  |
| Cheong et al. (2016) |  |  |  |  | ✓music therapy |  |  |  |  |  | ✓board certified music therapist |  | 2 | |  |
| Daykin et al. (2017) |  |  |  | ✓visitors could attend music sessions | ✓participatory music (reminiscence, song-writing, composing, singing, playing instruments) |  |  |  |  |  | ✓Musician trained to work with PwD |  | 3 | |  |
| Windle et al. (2018) |  |  |  | ✓carers were not required to take part although some chose to | ✓art viewing activity, art-making | ✓community art partners |  |  |  |  | ✓lead artist with prior expertise and training in art and dementia |  | 4 | |  |
